# Correlation between DPOAE I/O functions and pure-tone thresholds

**DOI:** 10.1590/S1808-86942011000600012

**Published:** 2015-10-19

**Authors:** Ualace de Paula Campos, Renata Mota Mamede Carvallo

**Affiliations:** 1 Specialist, PhD Student; 2 Associate Professor

**Keywords:** audiometry, hearing, otoacoustic emissions, spontaneous, hearing tests

## Abstract

**Abstract:**

Different studies have been carried out in order to correlate audiometric thresholds and distortion product otoacoustic emissions measurements (DPOAE). However, high variability and external interferences make hearing thresholds estimates by means of the DPOAE very little sensitive. The aim of this study was to check the correspondence between the pure tone thresholds and the cochlear response thresholds by DPOAE Input/output functions, considering the influence of the following variables: gender, past of acute otitis media, and ear side.

**Method:**

Prospective study comprehending 69 normal hearing individuals. Multiple mix regression models were applied to evaluate the correspondence between the two measurements studied.

**Results:**

Statistically significant positive correlation was observed among all the frequencies compared (2000, 3000, 4000 e 6000 Hz).

**Conclusions:**

The 1dB HL resolution pure tone thresholds and the above-mentioned variables had a direct impact on the high correlation between the measures studied, and it also reduced response variability. Nevertheless, response variability was still high, limiting the use of DPOAE I/O functions for hearing threshold estimates. We suggest that these variables should be considered for future studies with pure tone thresholds estimations by DPOAE I/O functions.

## INTRODUCTION

The healthy cochlea responds in a non-linear and compressive fashion to sound stimuli. The origin of non-linearity is on the basement membrane mechanisms. Compression implies that the cochlear amplifier gain in weak stimuli (<50 dB) is greater than the gain in moderate and strong stimuli (>50 and 80 dB, respectively). This cochlear amplification mechanism has as byproduct: the otoacoustic emission responses. The lack of compression and of non-linearity is associated with high audiometric thresholds, recruitment, reduction in frequency selectivity and poor temporal processing^1^.

Distortion product evoked otoacoustic emissions (DPOAE) are generated in response to the presentation of two pure tones (f1 and f2 stimuli), presented simultaneously. The response capture paradigm of the 2f1-f2 DPEOAs is the one most used, because it presents robust and reliable responses^2^. DPOAEs provide information about the function, the cochlear active mechanism and the very mobility of the outer hair cells (OHC)^3^.

Through the OAEs we can see the cochlear nonlinearity^4^, cochlear compression and the functioning of the OHC, which can be studied and quantified. The auditory mechanism is non-linear and the non-linearity of this mechanism is essential for a normal auditory function^5^. Because of all these characteristics, the OAE are broadly used in clinical practice and in research for the detection of hearing losses in different populations.

Through OAEs there is the possibility of assessing the otoacoustic emission growth curves, and the distortion product is the most used recording mode for this end. Today, this is the most used test for measuring and understanding cochlear non-linearity in humans^6^. According to Ruggero & Rich^7^, there is a linear growth of the DP-OHCOAE (Distortion Product Outer Hair Cell Otoacoustic Emissions) for weak stimuli (<50 dB), a non-linear growth for moderate stimuli (50 to 80 dB) and, again, a linear growth for intense stimuli (>80 dB).

Nonetheless, the high variability response makes the assessment by means of DPOAE very little sensitive. Mills et al.^8^ suggested that, for studies with DPOAE values, the definition of normal hearing should be more strict, more accurate. After assessing 20 young adults (18 to 24 years) with audiometric thresholds lower than 10dB and tympanometric curves with admittance peaks (226 Hz) between -30 and +30 of the AP, the researchers found a strong correlation between audiometric thresholds and the DPOAE, with a maximum variation of 13 dB in 95% of the correlations performed. The strict inclusion criterion in relation to the audiometric and tympanometric thresholds may have been the main reason behind our finding of such high correlation.

Gorga et al.^6^ controlled the external interferences with the goal of reducing the DPOAE response variability. Among other controls, the researchers created rules to finalize the exam and for the acceptance of responses only with a maximum background noise of 25 dB SPL.

Garner et al.^9^, also studied the DPOAE variability, and they stated that the frequencies with the highest variations in the DPOAE are those of 500; 1,000; 5,656 and 8,000 Hz. According to these researchers, such variability was caused by the background noise effect for the low frequencies, and by the transmission characteristics of the middle ear for the high frequencies. The DP-OHCOAE responses also presented a greater response variability to weak stimuli (30 dB) when compared to stimuli higher than 55 dB. Researchers suggested we strictly controlled the background noise, keeping the intensity level constantly low.

Job and Nottet^10^ and Yilmaz et al.^11^ studied the influence of past otitis media and DPOAE amplitude reduction when compared to individuals without the same background. Both concluded that the DPOAE study was a sensitive tool for the detection of subclinical dysfunctions, regardless of origin.

Many investigations considered the correlation between DP-OHCOAE and audiometric thresholds^4,^^12^, ^13^, ^14^, ^15^, ^16^. Boege & Janssen^12^ found a significant correlation between audiometric thresholds and the DP-OHCOAE. They also stated that the DPOAE reflect the functioning of the peripheral processing of sound and enables a reliable estimate of the audiometric thresholds for hearing losses up to 50 dB HL. Schmuziger et al.^14^ and Hatzopoulos et al.^16^ also found a high correlation between the two measures, even using different equipment. Despite the high correlation, both suggested that the use of growth curves is limited because of the high response variability. Probst & Schmuziger^17^ go beyond, stating that the benefits of this method to estimate audiometric thresholds are very likely limited.

So far, most of the attempts to correlate otoacoustic emissions were carried out using the OAE amplitude value and the audiometric threshold with a 5dB HL resolution. With the use of the DPOAE growth curve thresholds and 1dBHL resolution audiometric thresholds, we expect to find a positive correlation with low variability between the two values, depending on three variables: a past episode of acute otitis media, gender and right/left ear, in other words, it is expected that the three factors mentioned must be considered before the DP-OHCOAE and the audiometric thresholds are compared.

The goal of the present study was to check the correspondence between the tonal audiometric thresholds and the cochlear response thresholds obtained by means of the distortion product otoacoustic emission growth curves, considering the influence of the variables: gender, a past of acute otitis media and ear side.

## MATERIALS AND METHODS

This is a prospective, cross-sectional analytical study, assessed and approved by the Research Project Analysis Committee from our institution, with protocol number: 0086/08.

## SERIES

We had a total of 69 individuals (138 ears), 21 men and 48 women with ages between 18 and 32 years. All participants had audiometric thresholds lower than or equal to 25 dBHL in the frequencies of 250, 500, 1000, 2000, 3000, 4000, 6000 and 8000 Hz, besides type A curve tympanometry and acoustic reflexes present at 100 dBHL in the frequency of 1000 Hz. Exclusion criteria were: excess drinking of alcohol (one or more daily doses) and drugs (one or more used per week). Seven ears were taken off the study for presenting changes in the audiometric thresholds or tympanometry.

### Procedures

The assessment lasted for about 50 minutes per participant and all the assessments were carried out by the same examiner. After explaining the procedures and having the participants sign the informed consent form, the tests started in the following order: interview - in which the participants were asked about an episode of acute otitis media in the past 10 years, on the exclusion criteria and general health conditions; ear canal inspection, immittance measures [GSI33 middle ear analyzer (v2; Grason-Stadler)], with a 226 Hz probe frequency; threshold tonal audiometry [GSI 61 audiometer (Grason-Stadler, Madison, WI, USA)] in a sound-treated booth in the frequencies of 1000, 2000, 3000, 4000, 6000, 8000, 500 and 250 Hz, in order to determine the audiometric thresholds with a resolution of 1 dBHL after obtaining the threshold with a 5dBHL resolution and, finally, study of the DPOAE growth curve (ILO292 USB *version* 6) in a sound-treated booth for the frequencies of 2000, 3000, 4000 and 6000 Hz. We used the f_1_/f_2_=1.22 ratio and the “DPOAE=2f_2_-f_1”_ formula for the frequencies and there was a control for the maintenance of the background noise up to the maximum of -10 μPa. We used the paradigm proposed by Kummer et al.^18^ who suggests the formula: “L_1_ = (0.4 x L_2_)+39 dB SPL”, in order to establish the stimulus intensity. In such formula, L_1_ refers to the stimulus frequency at the f_1_ frequency, and L_2_ is related to the stimulus of frequency f_2_, in such a way that both stimuli are presented with unequal intensities. The DPOAE response curve studies was carried out with decreasing stimuli, starting at 75 dB SPL from L_2_ and 5dB SPL increments until the intensity reached the minimum intensity of 30 dB SPL. The ILO292 *version 6* equipment measuring unit for the DP-OHCOAE response amplitude value is the micro Pascal (μPa). The DP-OHCOAE thresholds were determined at the lowest stimuli intensities which generated an S/R ≥ 3 (μPa) ratio response above the background noise and, parallel to that the same presence criteria should happen for the stimuli responses in the immediately higher intensities.

### Statistical Analysis

We employed comparative and descriptive methods. P values lower than 0.05 were considered statistically significant.

The study of the association between audiometric thresholds and the DP-OHCOAE was adjusted by mixed multiple regression models because of the possible existence of the correlation in the measures done in the two ears of the same individual. The tonal audiometry threshold was considered as response variable, and as variables which could predict the DP-OAE amplitudes were their interactions with gender, past episodes of AOM and ear side, and its interactions. We adopted the backward procedure of variable selection. The inclusion of the variables model representing the interactions between the amplitude and gender, past episodes of AOM and ear side enabled us to assess whether the association between the audiometric threshold and the DP-OHCOAEs depend on these factors.

## RESULTS

Among the ears tested, 37 were from people with a past of acute otitis media in the past 10 years. All comparisons were carried out with tests which assessed the interactions between variables: ear, gender and past episodes of AOM. On Table 1 it is possible to see the number of ears from each “subgroup”.

**Table 1:** Subgroups established according to past of disease, gender and ear.

Past AOM	Gender	Ear	N
	F	R	33
	F	L	31
No	M	R	15
	M	L	15
	F	R	13
	F	L	12
Yes	M	R	6
	M	L	6

The audiometric threshold mean values varied between -1.18 dBHL at 250 Hz and 3.23 dBHL at 8000Hz. The audiometric thresholds did not suffer changes depending on any variable. The regression analysis was carried out in order to study the correlation between responses in the frequencies of 2000, 3000, 4000 and 6000 Hz. The 2000 Hz frequency presented mean thresholds of 1.18 dBHL with a standard deviation (SD) of 5.66 dBHL. At 3,000 Hz, the mean was of -0.38 with a SD of 6.5. At 4000 and 6000 Hz, the audiometric mean values were of 0.95 (SD=7.48) and 3.23 (SD=7), respectively.

**Figure 1:**
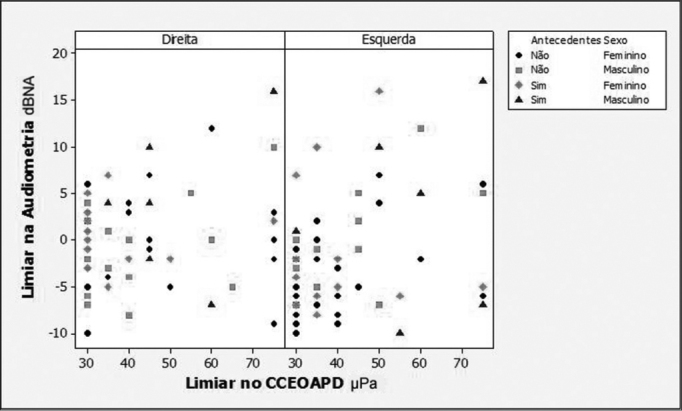
Scatter diagram of audiometric thresholds and of DPOAE growth curve thresholds, considering variables: ear, past episodes of otitis media and gender - 2000 Hz.

**Figure 2:**
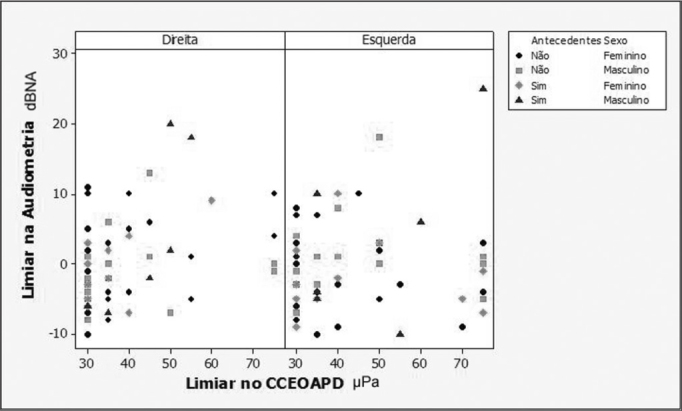
Scatter diagram of audiometric thresholds and of DPOAE growth curve thresholds, considering variables: ear, past episodes of otitis media and gender - 3000 Hz.

**Figure 3:**
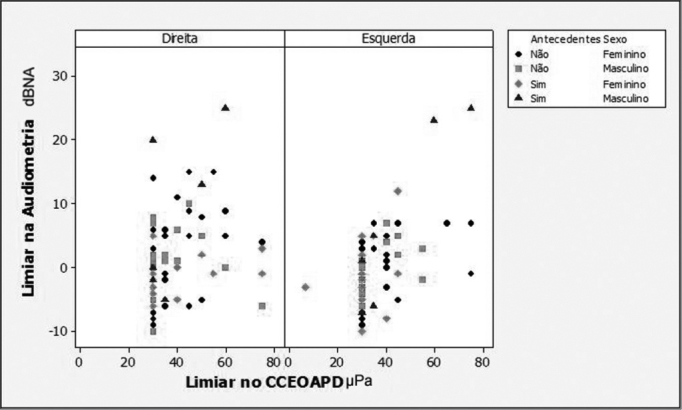
Scatter diagram of audiometric thresholds and of DPOAE growth curve thresholds, considering variables: ear, past episodes of otitis media and gender - 4000 Hz.

**Figure 4:**
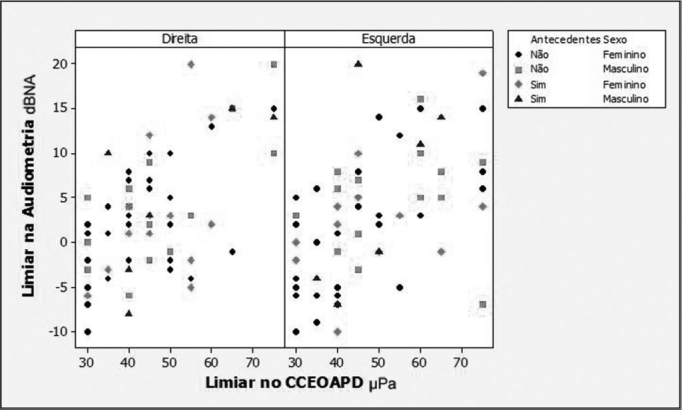
Scatter diagram of audiometric thresholds and of DPOAE growth curve thresholds, considering variables: ear, past episodes of otitis media and gender - 6000 Hz.

Regarding the association between audiometric thresholds and DP-OHCOAE thresholds, the regression models adjustments showed that there was a positive and statistically significant correlation in all the compared frequencies (2,000; 3,000; 4,000 and 6,000 Hz). Because of the background noise interference at 500, 1000 and 1500 Hz, it was not possible to correlate the lower frequencies, because there were a large number of absent responses in these frequencies. In Figures 1, 2, 3 and 4 we show the scatter diagrams, in which one can see the spread of values in which the regression models adjustments show a positive correlation in all frequencies tested.

In adjusting the regression model for 2000 Hz (Figure 1), we noticed that there was a positive correlation between audiometric thresholds and DP-OHCOAE thresholds (*p*=0.006); and the correlation did not depend on the gender (*p*=0.984), past AOM (*p*=0.517) and ear side (*p*=0.580). The linear coefficient did not depend on gender (*p*=0.143) or ear side (*p*=0.113) but it was greater in individuals with a past of AOM (*p*=0.034).

Since the regression adjustment showed a positive correlation between the measures studied, it was possible to calculate the linear and angular coefficient, which helped in the estimation of one measure for another. The estimates for the linear and angular coefficients were:

• Hearing threshold (without past AOM) = -6.0+0.1*DP-OHCOAE;
• Hearing threshold (with past AOM) = -3.3+0.1* DP-OHCOAE;

Where values -6.0 and -3.3 correspond to the linear coefficients and the value 0.1 corresponds to the angular coefficient.

Two estimation formulas were created, since the linear coefficient depends on past episodes of acute otitis media.

The maximum audiometric threshold variation between individuals was 25 dB HL, nonetheless, when we consider past episodes of middle ear disorders, the maximum variation was of 20 dBHL between individuals without past episodes of AOM who had DP-OHCOEA thresholds in 75 μPa; and 16 dBHL in individuals with past episodes who had DP-OHCOAE thresholds in 30 μPa.

In the frequency of 3000 Hz (Figure 2), the regression model adjustment had a positive correlation (*p*= 0.036) and the correlation did not depend on any variant (gender *p*=0.065; past AOM=0.194; ear side *p*=0.301). The adjusted model provided the following equation to estimate the threshold in this frequency:

• Hearing threshold = -3.9+0.09*DP-OHCOAE;

In this frequency, the maximum inter-individual response was 28 dB HL. Notwithstanding, when we consider past episodes of middle ear disorders and gender, the maximum variation was of 21 dBHL among the individuals without a past episode of AOM who had DP-OHCOAE thresholds of 30 μPa and 18 dBHL in the individuals with past episodes of AOM who had DP-OHCOAE thresholds in 50 μPa. Like in the 2000 Hz, the consideration about the presence or absence of past episodes of AOM reduced the variability of the estimations.

Results from the regression model for the 4000 Hz (Figure 3) frequency also pointed that there is a positive correlation between the two threshold measures, with a p-value of 0.002. The correlation did not depend on past episodes AOM (*p*= 0.314), nonetheless, it depended on gender (*p*=0.009) and ear side (*p*=0.033). The linear coefficient depended only on the ear side effect (*p*=0.028). The estimate considered variants: gender and ear side:

• Hearing threshold (RE/ Fem) = -4.1+0.11*DP-OHCOAE threshold;
• Hearing threshold (RE/ Male) = -4.1+0.20* DP-OHCOAE threshold;
• Hearing threshold (LE/ Fem) = -11.3+0.29* DP-OHCOAE threshold;
• Hearing threshold (LE/ Male) = -11.3+0.37* DP-OHCOAE threshold;

In the 4000 Hz frequency, the maximum inter-individual response variation was of 30 dB HL. When past episodes of middle ear disorders are considered, the maximum variation was of 25 dBHL for individuals without or with past episodes of AOM. If we consider ear side and gender effects, the maximum variation was reduced to 20 dB HL.

The 6000 Hz frequency (Figure 4) had a positive correlation in the regression model, non-gender dependent (*p*= 0.984), past episodes AOM (*p*= 0.326) and ear side (*p*=0.369). The linear coefficient did not depend on gender (*p*= 0.757), ear side (p=0.406) and past episodes of AOM (*p*= 0.733). The adjusted model was:

• Hearing threshold = -9.0+0.26*DP-OHCOAE;

The maximum inter-individual variation in the frequency of 6000 Hz was 25 dBHL among individuals with past episodes of AOM with DP-OHCOEA growth curve thresholds of 55μPa. Individuals with past episodes of AOM had a variation of 17 dB HL.

## DISCUSSION

Our goal here was to study to see if in normal hearing individuals the pure tone thresholds would be correlated with the distortion product otoacoustic emission threshold, considering the effects of right and left ear interference or that of gender and past episodes of acute otitis media in the last 10 years. The major difference between this study and previous ones^10^,^11^, was the way used to determine the electroacoustic threshold: through the DPOAE growth curve thresholds (see procedures) and not through the DP-Gram amplitude in a specific intensity. Results suggest a high correlation between the two measures. In relation to the factors which could influence the tests, it was only in the frequencies of 2000 and 4000 Hz that there were significant interferences from past episodes of acute otitis media (in 2000 Hz), gender and ear side (in 4000 Hz), and it was necessary to create separate formulas to estimate hearing thresholds in these frequencies according to the variables which affected them.

The audiometric threshold variability for a given DPOAE growth curve threshold, as seen on figures 1 through 4, was of up to 30dBHL (in 4000Hz), when variables gender, ear side and past episodes of otitis media in the last 10 years were considered. This high variability compromises the use of the procedure to estimate audiometric thresholds. Schmuziger et al.^14^ stated that the estimation of audiometric thresholds through the DPOAE has an inter-individual variation of up to 40 dB. They also stated that this variation very likely limits the clinical benefits for this method. However, in the present study, when one considers the variables gender, ear and, especially, past episodes of AOM, there was a reduction in audiometric threshold variability between 5 and 14 dB HL, reaching the values of 16 dBHL in 2000Hz and of 25dBHL in 6000Hz. The study of audiometric thresholds (tonal audiometry) with a 1dBHL resolution also contributes to the reduction of this variability.

Gorga et al.^6^ studied the non-linearity of the human cochlea in 500 and 4000Hz and suggested that there is a broader dynamics range and greater gain of the cochlear amplifier in 4000Hz, when compared to 500Hz. In the present study, the variations in the otoacoustic emission growth curve thresholds were greater in the frequencies of 4000 and 6000 Hz when compared to the 2000 Hz frequency. The greater variation in the high frequencies can be interpreted in two ways: First, the better functioning in high frequencies, suggested by Gorga et al.^6^ may have been influenced by the sequelae of past episodes of acute otitis media may increase middle ear mobility, which could mainly affect the high frequencies^19^. Therefore, the cochlear amplifier action may also be indirectly affected and it may be one of the factors which generate the greater variability of the frequency response of the equipment probe used in this study, above 5000 Hz, which may be the main cause of variability in the attainment of responses in 6000 Hz.

Gorga et al.^6^ also used rules for exam termination based on background noise stabilization in order to increase result reliability. In the present study, the maintenance of background noise stability at -10 μPa (included as exam termination criterion) and establishing DP-OHCOAE thresholds as the lowest L_2_ value with S/R ≥ 3 μPa ratio with the two subsequent L_2_ levels maintaining, at least, the same S/R ratio (as per described in the methods), have contributed to the high correlation between the two values. The correlation between this threshold and the audiometric thresholds was significant in all the frequencies compared. Unfortunately, because of the background noise in 500 and 1000 Hz, it was not possible to correlate the low frequencies; however, this is recurrent in studies which used the otoacoustic emission values.

The high variability in the responses may cause a considerable risk of false-positive responses, reducing DP-OHCOAE reliability. Boege and Janssen^12^ argued that the fact that the growth curves did not show a high correspondence with the audiometric thresholds may be due to modifications or changes unrelated with the audiometric thresholds or which are not detectable by them; and they also stated that these factors must be investigated. Gorga et al.^4^ also discussed the high variability of the DPOAE growth curves, assuming that some criterion must be included in the studies, such as no changes in the middle ear and the presence of DPOAE with L2=65 dB SPL. The inclusion of variables: gender, ear side and past episodes of otitis media increased the sample's homogeneity per subgroup and, consequently, reduced the variability of tonal threshold estimation, enabling the identification of mild differences between the behaviors of each subgroup.

The present study did not solve the issue of variability in detecting DPOAE growth curves; nonetheless, it presented a way to reduce them. Even then, the variability was very high, reaching 25 dB HL; however it was lower than those in studies which used only the lowest positive value of the growth curves^14^,^16^ or the values collected in the Dp-Gram^10^,^11^.

## CONCLUSIONS

There was a high and significant correlation between the audiometric thresholds and the lowest DPOAE intensity, in which the signal-to-noise ratio was of, at least, 3μPa with both intensities above showing the same relation. The study of the audiometric thresholds with a 1dBHL resolution and the separation of the participants in subgroups considering the variables: gender, past episode of acute otitis media and right/left ear, had a direct impact on the high correlation between the audiometric thresholds, the DPOAE growth curves and the reduction in response variability. We suggest that these variables must be considered in future studies of threshold estimation through DP-OHCOAE. Despite these conclusions, DPOAE growth curves do not replace the audiometric exam both because of variability and because of the structures which are assessed. DP-OHCOAE may contribute to an enhancement in diagnostic and prognostic accuracy in clinical decision making associated with the auditory system, but they are not able to accurately estimate the audiometric thresholds.

## ACKNOWLEDGEMENTS

FAPESP - Fundação de Amparo à Pesquisa do Estado de São Paulo for granting the PhD scholarship.
